# Sleeve Gastrectomy and Gastroesophageal Reflux Disease

**DOI:** 10.1155/2013/741097

**Published:** 2013-07-15

**Authors:** Michael Laffin, Johnny Chau, Richdeep S. Gill, Daniel W. Birch, Shahzeer Karmali

**Affiliations:** ^1^Department of Surgery, University of Alberta, Edmonton, AB, Canada; ^2^Center for the Advancement of Minimally Invasive Surgery (CAMIS), Edmonton, AB, Canada

## Abstract

Bariatric surgery, when combined with lifestyle and medical interventions, is a common and successful treatment modality in the obese patient. Laparoscopic sleeve gastrectomy is one such procedure that has increased in popularity as a definitive bariatric operation. Although laparoscopic sleeve gastrectomy has been shown to be effective in producing weight loss and improving type 2 diabetes mellitus, its effect on gastroesophageal reflux disease (GERD) has been inconsistent. This paper aims to summarize the available literature regarding GERD prevalence following laparoscopic sleeve gastrectomy, 8 studies demonstrate increased GERD prevalence, and 5 demonstrate decreased GERD prevalence following laparoscopic sleeve gastrectomy. The relationship between GERD and SG is complex and no clear relationship exists. The anatomic and physiologic changes caused by laparoscopic sleeve gastrectomy are discussed in the context of these inconsistent results.

## 1. Introduction

Obesity is a worldwide epidemic. In 2008 the World Health Organization estimated that more than 500 million adults are clinically obese (BMI > 30 kg/m^2^) worldwide [1]. Obese patients develop obesity-related comorbidities including type 2 diabetes mellitus (T2DM), hypertension, dyslipidemia, coronary artery disease, certain types of cancer, and gastroesophageal reflux disease (GERD) [2–6]. Healthcare providers must consider a treatment's effect on weight loss, as well as its impact on obesity-related comorbidities. Bariatric surgery, in conjunction with intensive lifestyle interventions and medical treatments, has been shown to produce marked weight loss and improvement in many obesity-related comorbidities [7]. Laparoscopic sleeve gastrectomy (SG) is effective in producing weight loss and improving T2DM [8]; however, evidence of SG's effect on GERD is inconsistent [9]. 

## 2. Gastroesophageal Reflux Disease (GERD) Background 

The Montreal Classification defines GERD as a condition that develops when reflux of stomach contents in the esophagus causes troublesome symptoms or complications [10]. Symptoms associated with GERD include heartburn, regurgitation, dysphagia, laryngitis, and chronic cough. Prolonged acid exposure within the esophagus can lead to histopathologic and structural changes such as peptic stricture and Barrett's esophagus. 

The fundamental inciting event in GERD is the retrograde movement of gastric contents into the esophagus [11]. A number of physiological factors increase the likelihood of this event, namely: transient lower esophageal sphincter relaxations [12–14], a hypotensive (or relatively hypotensive) lower esophageal sphincter (LES) [15], and anatomic disruption of the gastroesophageal junction (e.g., a hiatal hernia) [16–18]. The interplay between these elements has not been well elucidated, although it has been suggested that transient lower esophageal sphincter relaxations are responsible for mild symptoms, and more severe symptoms are due to the other two factors [19]. 

Transient lower esophageal sphincter relaxations, a hypotensive LES, and hiatal hernia are associated with obesity. Ayazi et al. demonstrated that increased weight was associated with both esophageal acid exposure and mechanical dysfunction of the LES [20]. Wu et al. reported an association between obesity and frequency of transient lower esophageal sphincter relaxation [21].

The diagnosis of GERD is imprecise as there is no gold standard [22]. Recently published practice guidelines state that a diagnosis of GERD can be established in the setting of typical symptoms [23]. Given the lack of gold standard in GERD diagnosis, evaluation of GERD includes a variety of modalities including 24-hour esophageal pH monitoring, esophageal manometry, endoscopy, and symptom reporting [24, 25]. Studies assessing GERD often use symptom reporting as a primary endpoint, though others focus on more objective measures such as esophageal pH monitoring.

## 3. Sleeve Gastrectomy 

SG was developed in 1988 as the initial procedure in a staged approach to patients with morbid obesity [26]. It was described as an isolated procedure in 1993 by Johnston et al. [27] with a minimally invasive alternative developed in 1999 [28]. SG has gained popularity since then as a definitive bariatric surgical procedure [29, 30].

SG involves resection of the greater curvature of the stomach and preservation of the pyloric valve and gastroesophageal junction. This maintains patency of the proximal alimentary tract and provides a restrictive and biochemical impetus for weight loss [31–33]. An advantage of SG compared to other bariatric procedures is that it does not involve an anastomosis. SG was initially considered a purely restrictive weight-loss procedure but there is newer evidence that alteration in gastrointestinal hormones plays a role. These changes are manifested by a decrease in endogenous ghrelin (a hormone associated with hunger) production [34] and decreased gastric and small bowel transit times [35].

## 4. Sleeve Gastrectomy and Gastroesophageal Reflux

Bariatric surgical procedures such as laparoscopic Roux-en-Y gastric bypass (RYGB) have been shown to improve symptoms related to GERD [36]. However, the effect of SG on GERD remains controversial. The current literature can be divided into two categories: those that demonstrate an increase in GERD prevalence after SG (Table 1) and those that demonstrate a decrease in GERD prevalence after SG (Table 2). 

### 4.1. SG Associated with an Increase in GERD Prevalence

The measured increase in GERD prevalence ranged from 2.1% to 34.9% in the analyzed literature. There was marked heterogeneity between the studies in regard to a number of factors including preoperative BMI, method of evaluating GERD, exclusion criteria, length of follow-up, and operative technique.

Two studies, Arias et al. [37] and Braghetto et al. [38], excluded patients with GERD preoperatively. Arias et al. [37], a single institution retrospective chart review conducted over 26 months, demonstrated a 2.1% incidence of GERD after SG. Braghetto et al. [38] conducted a single institution study that excluded patients with GERD preoperatively and reported an incidence of 27.5% after SG. Followup was not clearly reported and it is not stated whether the study was conducted prospectively. It is reasonable to assume that the modest 2.1% incidence of GERD cases reported by Arias et al. [37] would have been offset by a decrease in existing GERD if patients with GERD preoperatively were not excluded. The 27.5% increase reported by Braghetto et al. [38] may have been sufficient to cause an overall increase in GERD regardless of exclusion criteria. 

Both works of Carter et al. [39] and Howard et al. [41] were single institution retrospective chart reviews focused on the relationship between GERD and SG. Carter et al. [39] presented 176 patients and recorded weight loss at 6, 12, and 24 months. Data for GERD was presented in two categories: early (symptoms occurring in the first 30 days) and late (symptoms occurring after 30 days). Early post-SG GERD prevalence was increased 14.4% from the preoperative value, and late post-SG GERD was increased by 12.6%. Howard et al. [41] demonstrated a 14% increase with a mean follow-up time of 32 weeks.

Himpens et al. [40] was a retrospective single institution study that described a triphasic response to GERD. An initial increase at one year, followed by a decrease at three years, and an increase at six years. Other studies lack the follow-up necessary to assess for this third phase. For example, Melissas et al. [33], a single centre prospective study, showed the same initial two phases (an increase followed by a decrease at two years) but did not have a sufficient follow-up time to evaluate for a possible third peak in GERD prevalence. 

The work of Nocca et al. [43] was a multicenter prospective study that recruited 163 patients and demonstrated a 5.7% increase in prevalence after SG. The work of Tai et al. [44] was a prospective trial, but was performed at a single institution and a single surgeon conducted all operations. Preoperative BMI was the lowest among all presented studies at 36.3 kg/m^2^. This corresponded with a 34.9% increase in GERD prevalence, the largest increase in GERD prevalence of any studies listed.

The work of Lakdawala et al. [42] was a single institution retrospective study designed to compare SG with RYGB. It provided data that demonstrated a 4% increase in GERD prevalence after SG.

#### 4.1.1. Proposed Mechanisms for Increased GERD Prevalence after SG (Table 3)

The anatomy of the after SG stomach can predispose one to GERD. The angle of His, an anatomical feature protective against GERD [57], is disrupted. Himpens et al. [49] describe its destruction during SG as partially responsible for the increase in GERD in the first year after SG, and its restoration as responsible for the decrease in GERD seen by year three of their study. Volume and pressure assessments performed by Yehoshua et al. [50] have demonstrated decreased gastric compliance and increased gastric pressures after SG. Increased gastric pressure leads to a relative hypotension of the LES and may lead to increased reflux. 

A late anatomic change in SG patients called “neofundus” formation has been described by Himpens et al. [40]. Neofundus formation refers to the dilation of the sleeve to an extent where normal physiological compliance is exceeded. The dilation of the neofundus effectively creates a mid-stomach stenosis in SG patients. This may lead to gastric stasis and increased acid production.

Incompetence of sling fibres near the angle of His correlates with a decrease in LES tone [48, 58]; these fibres are often transected in sleeve gastrectomy. Hypotension of the LES after SG has been confirmed by Braghetto et al. [48] using esophageal manometry. 

Migration of the stomach into the chest (i.e., hiatal hernia) lessens the influence of the diaphragm on LES tone and leads to decreased LES pressures [59]. Baumann et al. [53] describe 10 of 27 patients developing intrathoracic sleeve migration after SG.

Hypomotility and dysmotility of the gastrointestinal tract may cause GERD. Decreased plasma ghrelin levels have been shown to cause gastrointestinal dysmotility in animal models and are seen after SG [51]. A decreased plasma ghrelin can then predispose patients to GERD [51]. Delayed gastric emptying leads to an increase in stomach volume and pressure. Eventually increasing pressure can overcome the LES and lead to reflux. At one year after SG Himpens et al. [49] demonstrated slowed gastric emptying. This stands in contrast to their own measurements in SG patients three years postoperatively as well as the results reported by Melissas et al. [32] and Braghetto et al. [35] at one year. 

### 4.2. Decreased GERD Prevalence after SG

GERD prevalence decreased between 2.8% and 20% in the analyzed literature. Like the papers demonstrating increased GERD prevalence, there was a significant amount of heterogeneity between the studies. The study with the largest decrease in GERD prevalence was Weiner et al. [47], a prospective single centre study. It demonstrated a 20% decrease in GERD prevalence. 

Melissas et al. [32, 33] were both single centre prospective studies. Melissas et al. [33] demonstrated a decrease in GERD after SG at its terminal followup (24 months) while its 6-month analysis demonstrated a 21.7% increase in prevalence. Melissas et al. [32] reported a 5% decrease in GERD prevalence after SG.

The works of Chopra et al. [45] and Rawlins et al. [46] were both recent retrospective single centre studies, which reported decreases in GERD prevalence after SG of 0.5% and 4.1%, respectively. 

Although the studies presented in Table 2 describe an overall decrease in GERD, all but Weiner et al. [47] encountered patients with de novo GERD following their operation. The decrease in prevalence was due to a greater resolution of GERD in patients with the disease before SG compared to development of new GERD in patients with no prior symptoms. 

#### 4.2.1. Proposed Mechanisms for Decreased GERD Prevalence after SG (Table 4)

Given that obesity has been shown to increase GERD [20], any weight loss associated with SG should reduce the effect of obesity on GERD symptoms. 

Delayed gastric emptying can cause increases in stomach volume and pressure; therefore, accelerated gastric emptying should decrease stomach pressures. Melissas et al. [54] and Shah et al. [55] provided evidence that SG speeds gastric emptying. In contrast, Himpens et al. [49] demonstrated slowed gastric emptying at one year after SG. The alteration of sleeve anatomy postoperatively may decrease GERD. Stretching of the sleeve returns it to a more physiological level of compliance than in the immediate postoperative period [34]. Increased compliance may lead to decreased gastric pressures. Tension on the gastric walls below the cardia should be reduced after SG according to LaPlace's law (a lower diameter leads to lower wall tension if pressure is maintained) [56]. Eventual restoration of the angle of His [49] can decrease GERD symptoms as the angle of His is an anatomical feature protective against GERD [57]. Lazoura et al. [52] demonstrated that certain sleeve shapes correlated with decreased reflux symptoms. The resection of fundus in SG results in removal of the majority of the stomach's parietal cells. Parietal cells are the major acid producing cells in the stomach [60] and their removal may decrease gastric acid production.

## 5. Hiatal Hernia, SG, and GERD

The relationship between hiatal hernia, SG, and GERD warrants discussion. Studies utilizing this combined technique were not included above, nor in the recent systematic analysis by Chiu et al. [9]. Results have been promising. Daes et al. [61], a prospective single centre study, utilized hiatal hernia repair concurrently with SG and had a resultant 47.5% decrease in GERD prevalence postoperatively at 6 and 12 months. Soricelli et al. [62] demonstrated no de novo cases of GERD in any of the 97 patients undergoing SG and hiatal hernia repair. Even in cases where no hiatal hernia repair was planned the dissection involved in SG can function to reduce any hiatal hernia via traction applied during routine dissection. This was evidenced by the fact that in Daes et al. [61] of the 65 HH diagnosed preoperatively 31 had been reduced through the dissection associated with SG at the time of operation. Recognition and planned repair of hiatal hernia concurrently with SG may be one of the major technical factors in reducing post-SG GERD in the future. 

## 6. Conclusion

Currently, the effect of SG on GERD remains controversial. In addition there remains marked heterogeneity among the studies assessing GERD following SG. Assessment using a common objective standard for the evaluation of GERD, such as 24-hour pH monitoring, is lacking. Differences in surgical technique among studies may also contribute to difficulty comparing GERD following SG in the literature. Randomized controlled trials are needed with both objective and subjective measures of GERD to clarify this controversy in the future. 

## Figures and Tables

**Table 1:** Summary of studies showing increased GERD after sleeve gastrectomy.

Study	Patients (*n*)	Evaluation of GERD	Preoperative GERD (%)	Postoperative GERD (%)	Follow-up (months)	Size of Bougie (Fr)
Arias et al. [37]	130	Symptom reporting	0	2.1 (*n* = 3)	24	40
Braghetto et al. [38]	167	Symptom reporting, manometry, 24-hour pH monitoring, and endoscopy	0	27.5 (*n* = 46)	Not reported	Not reported
Carter et al. [39]	176	Patient survey and symptom reporting	34.6	49% within 30 days 47.2% persisting more than 1 month	24	34
Himpens et al. [40]	40	Medication usage	20 (*n* = 8)	21.8% at 1 year (*n* = 7/32), 3.1% at 3 years (*n* = 1/32), 23% at 6 years	12, 36, and 72	34
Howard et al. [41]	28	Symptom reporting, medication usage, and UGI swallow	25 (*n* = 7)	39 (*n* = 11)	8–92 weeks	38
Lakdawala et al. [42]	50	Symptom reporting, and medication usage	5	9	12	36
Nocca et al. [43]	163	Symptom reporting	6.1 (*n* = 10)	11.8	24	36
Tai et al. [44]	67	Symptom reporting	12.1 (*n* = 8)	47 (*n* = 31, 5 persistent)	12	36

**Table 2:** Summary of studies showing reduced GERD after sleeve gastrectomy.

Study	Patients (*n*)	Evaluation of GERD	Preoperative GERD (%)	Postoperative GERD (%)	Follow-up (months)	Size of Bougie (Fr)
Chopra et al. [45]	174	Symptom reporting	13.7 (*n* = 24)	13.2 (*n* = 23, 6 new)	6–36	34
Melissas et al. [33]	14	Motility and symptom reporting	14 (*n* = 2)	35.7 (*n* = 5) at 6 months 7 (*n* = 1) at 24 months	6 and 24	Not reported
Melissaset al. [32]	23	Motility and symptom reporting	35 (*n* = 8)	30 (*n* = 7, 2 new)	12	34
Rawlins et al. [46]	49	Symptom reporting	30.6 (*n* = 15)	26.5 (*n* = 13, 7 persistent, 6 new)	60	26.4
Weiner et al. [47]	120	Symptom reporting	35 (*n* = 42)	15 (*n* = 18)	60	32–44

**Table 3:** Proposed mechanisms for an increase in prevalence of GERD symptoms afterSG.

Proposed mechanisms for increased GERD after SG	Reference
Hypotension of the lower esophageal sphincter	Braghetto et al. [48]
Blunting of the angle of His	Himpens et al. [49]
Decreased gastric compliance and volume (leading to increased gastric pressure)	yehoshua et al. [50]
Decreased gastric emptying	Himpens et al. [49], Melissas et al. [32]
Decreased plasma ghrelin (dysmotility)	Nahata et al. [51]
Gastric sleeve shape	Lazoura et al. [52]
Increase in hiatal hernia	Baumann et al. [53]
Neofundus	Himpens et al. [40]

**Table 4:** Proposed mechanisms for a decrease in prevalence of GERD symptoms afterSG.

Proposed mechanisms for decreased GERD after SG	Reference
Accelerated gastric emptying	Melissas et al. [54], Shah et al. [55]
Decreased abdominal obesity	Pandolfino et al. [17]
Increased long-term gastric compliance	Karamanakos et al. [34]
Restoration of the angle of His	Himpens et al. [49]
Decreased acid production	
Gastric sleeve shape	Lazoura et al. [52]
Decreased wall tension	Santoro [56]
